# Effects of ziprasidone and olanzapine on body composition and metabolic parameters: an open-label comparative pilot study

**DOI:** 10.1186/1744-9081-9-27

**Published:** 2013-07-19

**Authors:** Subin Park, Ki Kyoung Yi, Min-Seon Kim, Jin Pyo Hong

**Affiliations:** 1 Division of Child and Adolescent Psychiatry, Department of Psychiatry, Seoul National University Hospital, Seoul, South Korea; 2 Department of Psychiatry, Asan Medical Center, Ulsan University College of Medicine, Seoul, South Korea; 3 Department of Internal Medicine, Asan Medical Center, Ulsan University College of Medicine, 388-1 Pungnap-2dong, Songpa-gu, Seoul, 138-736, South Korea

**Keywords:** Ziprasidone, Olanzapine, Weight gain, Resting energy expenditure, Body composition

## Abstract

**Background:**

In contrast to olanzapine, ziprasidone has been reported to cause minimal or no weight gain. This study aimed to compare the effects of ziprasidone and olanzapine on weight, body composition, appetite, resting energy expenditure, substrate oxidation, and metabolic parameters in adults with schizophrenia or other psychotic disorders.

**Methods:**

Twenty adults with schizophrenia or other psychotic disorders were randomized 1:1 to ziprasidone 20–160 mg/day or olanzapine 5–20 mg/day for 12 weeks. The mean doses during the 12-week study period were 109(range: 65–140) mg/day for ziprasidone and 11.6(range: 8.2–15.5) mg/day for olanzapine. Body weight, appetite, body composition, resting energy expenditure, and metabolic parameters were measured before and after drug treatment. Outcome measurements before and after medication were compared, and ziprasidone- and olanzapine-treated patients were compared.

**Results:**

After 12 weeks, olanzapine-treated patients showed significant weight gain, particularly fat gain, with increased low density lipoprotein-cholesterol and decreased high density lipoprotein-cholesterol concentrations. In contrast, ziprasidone-treated patients showed no significant weight gain with increased high density lipoprotein-cholesterol concentration.

**Conclusions:**

Ziprasidone was associated with a lower propensity for weight gain and central fat deposition than olanzapine. Studies in larger patient samples are required to confirm these results.

## Background

Weight gain is a common adverse effect associated with atypical antipsychotic drug treatment of patients with psychotic disorders [1-6]. Among these atypical antipsychotic drugs, clozapine and olanzapine are associated with the greatest weight gain [1,6]. This weight gain is a frequent cause of poor adherence to antipsychotic medications, as well as increased risks of metabolic complications, including obesity, dyslipidemia, glucose intolerance, and diabetes mellitus [7-11].

In contrast to other atypical antipsychotic agents, ziprasidone, a novel antipsychotic agent with a unique receptor-binding profile, has been reported to cause minimal or no weight gain [2,12-17]. This drug also has other significant metabolic advantages, including decreased risks of developing insulin resistance and dyslipidemia, compared with other atypical antipsychotics [14,18-20].

Several preclinical and clinical studies have provided insights into the mechanism underlying antipsychotic-induced weight gain. To date, increased appetite, sedation, and hyperprolactinemia have all been associated with antipsychotic-induced weight gain [21-24]. However, their precise mechanism has yet to be elucidated. Body weight is determined by the balance between caloric intake and energy expenditure, with a positive energy balance inducing fat deposition and weight gain. Several studies have therefore investigated the effects of atypical antipsychotic drugs, in particular olanzapine, on body composition and energy balance. Our recent preclinical study suggested that a 7-week treatment regimen of ziprasidone induced a significant decrease in weight gain in female rats, by increasing resting energy expenditure without decreasing food intake [25].

Although central fat deposition and dyslipidemia have been consistently reported in patients taking olanzapine [1,18,26-28], studies of the effects of olanzapine on resting energy expenditure [27,29,30] have yielded inconsistent results. Moreover, in contrast to the many studies of the metabolic effects of olanzapine, there have been few studies in humans on the effects of ziprasidone on body composition and energy expenditure.

Comprehensive comparisons of the metabolic effects of ziprasidone, the antipsychotic drug with a low propensity for weight gain, and olanzapine, a drug with a high propensity for weight gain, may be helpful in elucidating the mechanisms underlying weight gain and metabolic changes associated with antipsychotic drug use. We therefore compared the effects of 12 weeks of treatment with ziprasidone or olanzapine on weight, body composition, appetite, resting energy expenditure, substrate oxidation, and metabolic parameters in adults with schizophrenia and or other psychotic disorders.

## Methods

### Subjects

Adult patients who visited the Asan Medical Center experiencing a new onset psychotic episode (either first onset or a new onset discontinuation-related episode) were asked to participate in an investigation of the metabolic impact of olanzapine and ziprasidone. Patients were included if 1) they were 18–65 years of age, 2) had been diagnosed by a psychiatrist with a brief psychotic disorder, schizophreniform disorder, schizophrenia, or schizoaffective disorder according to Diagnostic and Statistical Manual of Mental Disorders, Fourth Edition (DSM-IV) criteria, and 3) had no other active illness. Patients were excluded if they 1) had a comorbid alcohol or drug abuse disorder, 2) were pregnant or lactating, 3) required treatment with medications other than lorazepam, clonazepam, zolpidem, benztropine, or propranolol for any medical or other psychiatric condition, or 4) had been administered any antipsychotic drugs during the previous three months. The study was approved by the institutional review board (IRB) for human subjects at the Asan Medical Center, and all subjects provided written informed consent.

### Procedures

The screening assessment included a full psychiatric history, a medical history, demographic data, and physical examination. We used the Korean version of the Structured Clinical Interview for DSM-IV [31] to determine psychiatric diagnosis and eligibility for this study. Following screening and enrollment, patients were randomized to receive ziprasidone or olanzapine for 12 weeks. Randomization was stratified by sex and age.

Within 14 days of screening, subjects were admitted to a psychiatric ward of Asan Medical Center for at least 24-hours. Before starting medication, subjects’ body weight, height and waist and hip circumference were measured; we also assessed their body composition, resting energy expenditure, and respiratory quotient. Fasting blood samples were taken for assays and appetite was measured. We utilized the Korean version of the Positive and Negative Syndrome Scale (PANSS) [32] to assess the severity of psychiatric symptoms in each patient.

Patients were subsequently started on 40 mg/day ziprasidone or 10 mg/day olanzapine. Dosage was adjusted depending on each patient's symptom severity and drug tolerability. Throughout the study period, the doses of ziprasidone ranged between 20 and 160 mg/day and those of olanzapine between 5 and 20 mg/day. The mean daily doses during the 12-week study period were 109(range: 65–140) mg/day for ziprasidone and 11.6(range: 8.2–15.5) mg/day for olanzapine.

After six weeks of treatment, subjects came to the psychiatry outpatient department of the Asan Medical Center. At this time, adverse events and medication use were assessed, and weight, height and waist and hip circumference were measured.

After 12 weeks of treatment, subjects were admitted as inpatients to the Asan Medical Center for 24 hours. In addition to the same assessments taken at 4 weeks, we assessed body composition, resting energy expenditure, respiratory quotient, PANSS, and appetite, and fasting blood samples were taken for assays.

### Outcome measurements

During their first and last inpatient visits, subjects were fasted for 12 h, starting the evening before, with minimum physical activity on the test day, and anthropometric measurements and blood samples taken upon awakening. Measurements taken during 6-week outpatient visits were taken at various times of the day.

Each subject completed questionnaires to assess hunger, fullness, desire to eat and prospective food consumption using subject appetite scores [33]. Ratings were made on a 10 cm Visual Analog Scale with words anchored at each end, expressing the most positive (e.g. good, pleasant) and most negative (e.g. bad, unpleasant) ratings. Mean appetite scores were calculated, with higher scores indicating a greater subjective appetite.

Body weight was measured with subjects wearing light clothing on a digital scale that was calibrated monthly. Height was assessed using a wall-mounted stadiometer. Waist circumference was measured at the umbilicus and hip circumference at the fullest part of the hips.

Body composition (fat mass, fat-free soft tissue mass, and muscle mass) was assessed by bio- electrical impedance using an InBody 720 body composition analyzer (BioSpace).

Substrate utilization (respiratory quotient) and resting energy expenditure were determined by indirect calorimetry with a MedGraphics CPX Ultima (Medical Graphics Corp, St Paul, MN) respiratory system. Oxygen consumption and carbon dioxide production were measured at 30-second intervals over 20 minutes at standard temperature, pressure, and humidity. Resting energy expenditure was calculated using the equation: Expenditure = (3.815 + 1.232 x VO_2_/VCO_2_) x VO_2_.

Standard laboratory methods were used to determine fasting concentrations of glucose, insulin, total cholesterol, high density lipoprotein cholesterol (HDL-C), low density lipoprotein cholesterol (LDL-C), triglycerides, C-peptide and prolactin.

### Statistical analysis

The study’s main outcome measures were changes in body weight, appetite, body composition, energy expenditure, respiratory quotient, and metabolic parameters. Due to the skewed distribution of outcome variables and small sample size, nonparametric statistical calculations were performed. Thus, median rather than mean changes were calculated. The Wilcoxon signed rank test was used to test whether outcome measures at last observation were significantly different from initial observations in each group. The Kruskal-Wallis H test was used to test whether the ziprasidone and olanzapine groups differed in percent changes of outcome measures from first to last observations. All statistical analyses were performed using SPSS (version 12.0; SPSS Inc., Chicago, IL), with statistical significance defined as an alpha level < 0.05. For the outcome variables, of which baselie measurements were significantly different between groups, R 2.14.0, Package [fANCOVA] was used to test whether the ziprasidone and olanzapine groups differed in absolute changes of outcome measures after adjustment for the baseline measures.

## Results

### Patient demographics

Each group consisted of 10 subjects (five males and five females), with similar median ages in the ziprasidone (34.50 years, interquartile range [IQR] = 26.25–40.25 years) and olanzapine (31.50 years, IQR = 26.50–41.25 years) groups (p > 0.99) and similar median baseline symptom severity, as measured by the PANSS, for patients treated with ziprasidone (67.50, IQR = 62.25–75.25) and olanzapine (82.00, IQR = 64.25–86.25) (p = 0.161). Median PANSS scores after 12 weeks of treatment were also similar in the ziprasidone (53.00, IQR = 42.00–78.75) and olanzapine (60.00, IQR = 53.00–67.00) groups (p = 0.677). Baseline measurements of outcome variables were not significantly different between groups (p > 0.10), except resting energy expenditure normalized to lean body mass (LBM) (p = 0.041).

### Body weight, appetite, and body composition

Table 1 showed that body weight, body mass index, and waist to hip ratio increased significantly in patients treated with olanzapine, but not ziprasidone. After 12 weeks, the median increase in body weight in the olanzapine group was 6.25 kg (IQR = 5.70–6.90 kg), representing an increase of 10.35% from first to last observation (p = 0.005), whereas the median weight gain in the ziprasidone group was 2.00 kg (IQR = 0.25–5.93 kg), representing an increase of 3.43% from first to last observation (p = 0.168). BMI increased significantly in olanzapine-treated patients, with a median increase of 2.10 kg/m^2^ or 10.93% of initial BMI (p = 0.005), but did not change significantly in the ziprasidone group, with a median increase of 0.80 kg/m^2^ or 4.09% of BMI (p = 0.139). Waist to hip ratio increased significantly in the olanzapine (median 0.03 or 3.62%, p = 0.005), but not in the ziprasidone (median 0.00%) group.

**Table 1:** Body weight, body composition, and appetite of ziprasidone- and olanzapine-treated patients (n = 10 per group)

	**Ziprasidone start**	**Ziprasidone end**		**Olanzapine start**	**Olanzapine end**		**Ziprasidone change (%)**^**a**^	**Olanzapine change (%)**^**a**^	
Measure	Median (IQR)	Median (IQR)	P^b^	Median (IQR)	Median (IQR)	P^b^	Median (IQR)	Median (IQR)	P^c^
BW(kg)	58.75 (54.28, 61.68)	60.85 (56.40, 67.88)	0.168	57.70 (51.25, 65.90)	65.70 (56.73, 72.88)	0.005^*^	3.43 (0.61, 9.20)	10.35 (9.27, 14.65)	0.016^*^
BMI (kg/m^2^)	21.50 (19.20, 24.00)	22.35 (19.48, 25.18)	0.139	21.85 (17.90, 23.65)	23.95 (20.35, 26.53)	0.005^*^	4.09 (0.55, 10.20)	10.93 (9.02, 15.08)	0.019^*^
Waist to hip ratio	0.86 (0.83, 0.91)	0.84 (0.82, 0.94)	0.916	0.85 (0.81, 0.88)	0.87 (0.85, 0.93)	0.005^*^	0.00 (−0.01, 0.02)	3.62 (2.16, 5.10)	0.004^*^
LBM (kg)	44.65 (38.13, 48.30)	46.35 (42.48, 51.20)	0.028^*^	44.35 (40.23, 54.30)	47.70 (41.63, 54.25)	0.074	3.05 (1.36, 9.71)	3.78 (−2.41, 11.53)	0.821
Muscle mass (kg)	41.90 (35.80, 45.68)	43.55 (39.88, 49.00)	0.028^*^	41.75 (37.95, 51.55)	43.80 (39.20, 51.50)	0.074	3.01 (0.53, 9.67)	3.90 (−2.50, 7.40)	0.762
Fat mass (kg)	17.05 (7.58-21.88)	14.65 (8.40, 23.23)	0.721	13.00 (10.15, 15.73)	17.70 (12.05, 20.33)	0.017^*^	7.84 (−12.34, 28.88)	31.57 (20.15, 56.03)	0.049^*^
Appetite	5.00 (4.31, 6.44)	5.00 (3.38, 5.31)	0.212	5.00 (4.19, 5.25)	5.00 (4.69, 6.13)	0.399	−20.0 (−29.80, 5.00)	0.00 (−6.25, 39.34)	0.059

*IQR* Interquartile Range, *BW* Body Weight, *BMI* Body Mass Index, *LBM*, *LBM*.

^a^, $\frac{\left(\mathrm{posttreatment}-\mathrm{baseline}\right)}{\mathrm{baseline}}$* 100; ^b^,Wilcoxon signed rank test; ^c^, Kruskal-Wallis H test; ^*^, p < 0.05.

The Kruskal-Wallis H test showed that, after 12 weeks of treatment, the percent changes in body weight (p = 0.016), BMI (p = 0.019), and waist to hip ratio (p = 0.004) were significantly greater in patients treated with olanzapine than with ziprasidone (Table 1). These between-group differences in changes in body weight (p = 0.023), BMI (p = 0.021), and waist to hip ratio (p = 0.009) were also significant after 6 weeks.

Subjective appetite did not differ significantly before and after treatment in both groups, but ziprasidone-treated patients tended to show a greater percent change in appetite than olanzapine-treated patients (−20% vs. 0%, p = 0.059) (Table 1).

As shown in Table 1, fat mass increased significantly in the olanzapine group, with a median increase of 13.00 kg or 31.57% of initial body fat (p = 0.017), but was not significantly changed in the ziprasidone group, with a median increase of 0.65 kg or 7.84% (p = 0.721). In contrast, LBM and muscle mass increased significantly in the ziprasidone group (p = 0.028 each), but not in the olanzapine group (p = 0.074 each), despite the significant increase in total body weight in the latter. The Kruskal-Wallis H test showed that the percent change in fat mass was significantly higher in olanzapine than in ziprasidone-treated patients (31.57% vs. 7.84%, p = 0.049), but that percent changes in LBM (3.78% vs. 3.05%, p = 0.821) and muscle mass (0.90% vs. 3.01%, p = 0.762) were similar.

### Energy expenditures

Table 2 showed that absolute 24-hour resting energy expenditure, resting energy expenditure normalized to LBM, and respiratory quotient were not significantly changed with olanzapine therapy. In contrast, ziprasidone-treated patients showed a significant increase in resting energy expenditure normalized to LBM, 6.91 kcal/g or 37.78%, without significant changes in absolute 24-hour resting energy expenditure and respiratory quotient. However, there were no statistically significant group-differences in the percent changes in energy expenditure measures. In addition, after adjusting for baseline resting energy expenditure normalized to LBM that had been different between two groups, there was no significant difference in changes in resting energy expenditure normalized to LBM between ziprasidone-treated and olanzapine-trated patients (4.93 vs. 1.50, t = 4.98, p = 0.627).

**Table 2:** Energy expenditure and respiratory quotients of ziprasidone and olanzapine-treated patients (n = 10 per group)

	**Ziprasidone start**	**Ziprasidone end**		**Olanzapine start**	**Olanzapine end**		**Ziprasidone change (%)**^**a**^	**Olanzapine change (%)**^**a**^	
Measure	Median (IQR)	Median (IQR)	P^b^	Median (IQR)	Median (IQR)	P^b^	Median (IQR)	Median (IQR)	P^c^
Absolute REE (kcal/24 hr)	1075.20 (1024.78, 1416.81)	1132.38 (1006.71, 1723.06)	0.678	1630.24 (1079.23, 1898.10)	1255.40 (1069.48, 1747.83)	0.241	1.41 (−11.98, 61.96)	−5.27 (−33.37, 10.58)	0.369
REE normalized to LBM (kcal/g)	18.29 (16.68, 22.39)	24.47 (22.48, 37.13)	0.011^*^	27.36 (19.91, 35.36)	27.60 (23.46, 34.85)	0.445	37.78 (18.36, 83.14)	17.17 (−17.47, 36.36)	0.072
Respiratory Quotient	0.82 (0.76, 0.88)	0.84 (0.80, 0.92)	0.262	0.86 (0.81, 1.01)	0.87 (0.82, 0.92)	0.574	5.81 (−3.33, 14.40)	−4.64 (−13.31, 13.27)	0.288

*IQR* Interquartile Range, *REE* Resting Energy Expenditure, *LBM*, *LBM*.

^a^, $\frac{\left(\mathrm{posttreatment}-\mathrm{baseline}\right)}{\mathrm{baseline}}$* 100; ^b^,Wilcoxon signed rank test; ^c^, Kruskal-Wallis H test; ^*^, p < 0.05.

### Laboratory findings

Table 3 showed that median LDL-C (17.00 mg/dl, p = 0.017) and prolactin (3.90 mg/dl, p = 0.028) concentrations increased significantly in olanzapine-treated, but not in ziprasidone-treated, patients. In contrast, median total cholesterol (7.50 mg/dl, p = 0.021) and HDL-C (4.00 mg/dl, p = 0.036) concentrations increased significantly in the ziprasidone, but not in the olanzapine, group. Other laboratory values, including glucose, insulin, and C-peptide did not change significantly in either group. The Kruskal-Wallis H test showed that the percent change in HDL-C concentration was significantly greater in the ziprasidone than in the olanzapine group (7.36% vs. −13.24%, p = 0.008), but that the percent changes of other laboratory values did not differ significantly between these two groups.

**Table 3:** Fasting laboratory parameters of ziprasidone and olanzapine-treated patients (n = 10 per group)

	**Ziprasidone start**	**Ziprasidone end**		**Olanzapine start**	**Olanzapine end**		**Ziprasidone change (%)**^**a**^	**Olanzapine change (%)**^**a**^	
Measure	Median (IQR)	Median (IQR)	P^b^	Median (IQR)	Median (IQR)	P^b^	Median (IQR)	Median (IQR)	P^c^
Cholesterol (mg/dl)	153.50 (143.00, 175.00)	171.00 (154.00, 185.25)	0.021^*^	176.50 (154.40, 205.75)	200.00 (160.00, 223.50)	0.203	4.95 (1.99, 24.55)	15.39 (−7.49, 22.09)	>0.99
Triglyceride (mg/dl)	89.00 (63.00, 112.75)	88.50 (81.00, 154.25)	0.114	73.50 (58.00, 106.50)	118.00 (71.50, 180.75)	0.086	30.35 (−3.72, 58.04)	62.54 (−3.08, 137.66)	0.290
HDL-C (mg/dl)	49.50 (44.00, 54.25)	58.50 (52.50, 61.50)	0.036^*^	59.00 (47.00, 65.25)	50.00 (46.75, 55.75)	0.092	7.36 (1.19, 29.55)	−13.24 (−23.56 , -4.02)	0.008^*^
LDL-C (mg/dl)	85.00 (71.55, 104.95)	100.00 (81.50, 109.00)	0.144	106.00 (77.50, 115.00)	129.00 (116.50, 143.00)	0.043^*^	7.07 (−0.68, 19.31)	16.04 (3.76, 84.25)	0.347
Glucose (mg/dl)	90.50 (81.75, 95.50)	95.50 (86.75, 98.00)	>0.99	89.50 (83.00, 96.50)	98.00 (87.75, 108.50)	0.241	−1.08 (−6.89, 6.69)	4.58 (−3.38, 17.92)	0.496
Insulin (IU/ml)	9.55 (5.58, 13.18)	6.30 (3.80, 10.35)	0.169	9.25 (5.85, 11.95)	6.75 (3.73, 10.50)	0.333	−25.46 (−47.37, 15.95)	−27.35 (−59.38, 39.37)	0.705
C-peptide (ng/ml)	2.30 (1.15, 2.45)	1.95 (1.25, 2.25)	0.357	1.70 (1.65, 2.33)	2.15 (1.35, 3.23)	0.314	−11.01 (−14.13, 15.56)	8.06 (−18.20, 89.71)	0.273
Prolactin (ng/ml)	9.55 (5.58, 13.18)	7.35 (6.15, 43.30)	0.285	10.95 (7.18, 19.38)	19.10 (11.33, 28.10)	0.028^*^	31.40 (−45.21, 262.32)	28.06 (−8.06, 122.40)	0.880

*IQR* Interquartile Range, *HDL*-*C* high density lipoprotein cholesterol, *LDL*-*C* low density lipoprotein cholesterol.

^a^, $\frac{\left(\mathrm{posttreatment}-\mathrm{baseline}\right)}{\mathrm{baseline}}$* 100; ^b^,Wilcoxon signed rank test; ^c^, Kruskal-Wallis H test; ^*^, p < 0.05.

## Discussion

We have shown here that patients treated with olanzapine for 12 weeks experienced significant weight gain, particularly fat gain, with increased LDL-C and decreased HDL-C concentrations, whereas patients treated with ziprasidone for 12 weeks experienced a non-significant weight gain, particularly muscle weight gain, with increased HDL-C concentration. To our knowledge, this is the most comprehensive metabolic outcome study of ziprasidone treatment, including resting energy expenditure and body composition, as well as anthropometric and metabolic parameters.

Weight gain was experienced by all but two subjects, both in the ziprasidone group, who were receiving 80 mg/day and 120 mg/day ziprasidone, respectively. The two subjects who experienced the greatest weight gain (10 kg over 12 weeks) included one receiving 80 mg/day ziprasidone and one receiving 10 mg/day olanzapine, the median respective doses in these patients. We observed no evidence of associations between weight gain and drug dose. However, the study sample was too small to determine such associations.

Consistent with previous results [26,27], we found that the significant weight gain in the olanzapine group was due primarily to an increase in body fat, accounting for about 61% of the total weight gain. Furthermore, the increase in the waist to hip ratio of olanzapine treated patients suggests central fat deposition. In contrast, the non-significant weight gain observed in ziprasidone treated patients was due primarily to an increase in muscle mass, accounting for about 75% of the total weight gain.

The fat gain observed in olanzapine-treated patients suggests that these subjects maintained a positive energy balance and deposited this energy in the form of triglycerides in adipose tissue. A positive energy indicates increased caloric intake or decreased energy expenditure [34]. Although we did not directly measure the caloric intake of these patients, we assessed their subjective appetite, which was expected to be associated with caloric intake. Previous studies have consistently reported that increased appetite is a common adverse effect of olanzapine treatment [26,28,29], but not of ziprasidone treatment [12,13,15,17]. Although we found that neither agent increased appetite significantly, ziprasidone-treated patients showed a greater decrease in appetite than olanzapine-treated patients, suggesting that this difference in change in appetite may have contributed, at least in part, to the lower propensity toward weight gain with ziprasidone than with olanzapine.

Consistent with previous studies in rats [35-37] and humans [26,27,29,30], olanzapine neither increases nor decreases resting energy expenditure. To our knowledge, no previous study has analyzed the effects of ziprasidone on resting energy expenditure. Although resting energy expenditure normalized to LBM increased significantly after treatment of ziprasidone, there were no statistically significant differences in the percent or absolute changes in energy expenditure measures between ziprasidone- and olanzapine- treated patients.

We measured respiratory quotient to determine whether ziprasidone or olanzapine affects substrate oxidation. We found that neither ziprasidone nor olanzapine affected respiratory quotient. In contrast, others [27] have reported an increase in respiratory quotient, indicating a shift in nutrient utilization away from fatty acids toward carbohydrates, after 12 weeks of olanzapine therapy, suggesting that this decrease in fatty acid oxidation may be associated with olanzapine-induced fat deposition and weight gain. Actually, the decision to utilize fatty acids or carbohydrates as fuels is highly regulated and depends on numerous exogenous and endogenous factors, including the level of caloric intake, the composition of food eaten, the size of glycogen stores, and the amount of adipose tissue [38]. Since we did not measure the amount or pattern of food intake, the interpretation of our results on respiratory quotient is limited.

Atypical antipsychotics, particularly olanzapine, have been associated with impaired glucose tolerance and type 2 diabetes [8-10]. None of our subjects developed diabetes, nor did we observe significant changes in the concentrations of fasting glucose, insulin, and C-peptide. However, consistent with previous findings [18,27,28], we found that olanzapine-treated patients exhibited adverse changes in several metabolic parameters, including tendencies for HDL-C and triglyceride concentrations to decrease and significant increases in LDL-C and prolactin concentrations. In contrast, ziprasidone-treated patients showed a significant increase in HDL-C concentrations and no changes in triglyceride, LDL-C, and prolactin concentrations. The increased cholesterol concentration we observed in ziprasidone-treated patients was not consistent with previous results, reporting that ziprasidone had favorable metabolic effects [14,18]. However, our comparison of ziprasidone- and olanzapine-treated patients showed no between-group difference in percent change in cholesterol concentrations.

We must interpret the present study's results in the context of the following limitations. First, owing to our small sample size, study findings should be considered preliminary in nature and require careful interpretation. Second, neither we nor the patients were blind to each subject's medication. Therefore there is a possibility of potential bias when we measured symptom severity by the PANSS and the patients estimated their appetite. However, most outcomes are obtained objectively from physical measurement or laboratory assay, which may not be biased due to no blindness. Finally, we did not measure caloric intake and levels of physical activity, and the use of sedatives was not controlled, all of which may have influenced the metabolic outcomes.

## Conclusions

In conclusion, we found that ziprasidone was associated with a lower propensity for weight gain and central fat deposition than olanzapine. Additional studies, in larger patient samples, as well as reliable measurements of food intake and activity level, are needed.

## Abbreviations

DSM-IV: Diagnostic and statistical manual of mental disorders, Fourth edition; PANSS: Positive and negative syndrome scale; HDL-C: High density lipoprotein cholesterol; LDL-C: Low density lipoprotein cholesterol; IQR: Interquartile range; LBM: Lean body mass; BMI: Body mass index.

## Competing interests

This work was supported by Pfizer Pharmaceuticals Korea. Dr. Jin Pyo Hong was the principal investigator in this study. Drs. Subin Park, Ki Kyoung Yi, and Min-Seon Kim have no conflicts of interests or financial ties to disclosure.

## Authors’ contributions

JPH designed the study. JPH, SP and KKY participated in data collection. SP and KKY analyzed the data and prepared the first draft of the report. JPH and MSK supervised the statistical analysis and interpreted the results. SP wrote the final report with input from all the authors. All authors had full access to all the data in the study and approved the version to be published.
